# Generation of resolving memory neutrophils through pharmacological training with 4-PBA or genetic deletion of TRAM

**DOI:** 10.1038/s41419-022-04809-6

**Published:** 2022-04-13

**Authors:** RuiCi Lin, Ziyue Yi, Jing Wang, Shuo Geng, Liwu Li

**Affiliations:** 1 Department of Biological Sciences, Virginia Tech, Blacksburg, VA 24061 USA; 2 Graduate Program of Genetics, Biotechnology and Computational Biology, Virginia Tech, Blacksburg, VA 24061 USA

**Keywords:** Innate immune cells, Inflammation

## Abstract

Neutrophils are the dominant leukocytes in circulation and the first responders to infection and inflammatory cues. While the roles of neutrophils in driving inflammation have been widely recognized, the contribution of neutrophils in facilitating inflammation resolution is under-studied. Here, through single-cell RNA sequencing analysis, we identified a subpopulation of neutrophils exhibiting pro-resolving characteristics with greater *Cd200r* and *Cd86* expression at the resting state. We further discovered that 4-PBA, a peroxisomal stress-reducing agent, can potently train neutrophils into the resolving state with enhanced expression of CD200R, CD86, as well as soluble pro-resolving mediators Resolvin D1 and SerpinB1. Resolving neutrophils trained by 4-PBA manifest enhanced phagocytosis and bacterial-killing functions. Mechanistically, the generation of resolving neutrophils is mediated by the PPARγ/LMO4/STAT3 signaling circuit modulated by TLR4 adaptor molecule TRAM. We further demonstrated that genetic deletion of TRAM renders the constitutive expansion of resolving neutrophils, with an enhanced signaling circuitry of PPARγ/LMO4/STAT3. These findings may have profound implications for the effective training of resolving neutrophils with therapeutic potential in the treatment of both acute infection as well as chronic inflammatory diseases.

## Introduction

Neutrophil serves as the first line of defense against invading pathogens as well as sterile inflammatory stimulants. While the inflammatory roles of neutrophils have been well recognized and extensively studied [1], there are less-understood yet highly crucial homeostatic roles of neutrophils in actively facilitating inflammation resolution and tissue repair [2, 3]. Resolving neutrophils can ameliorate inflammation by synthesizing pro-resolving lipid mediators, including Resolvin D1 and E1 [4, 5]; secreting microparticles carrying pro-resolving proteins [6]; expressing anti-inflammatory receptors such as CD200R and IL-1 receptor antagonist [7, 8] as well as soluble mediators, including SerpinB1 [9]; communicating with adaptive immunity and maintaining immune hemostasis via CD86; as well as promoting macrophage efferocytosis to expedite healing processes [10–12]. However, a clear characterization of resolving neutrophils and underlying mechanisms for their generation are currently lacking.

Although neutrophils were originally considered as short-lived effector cells adopting limited sets of defined effector functions, emerging studies suggest a much more complex scenario with the adaptability and memory of neutrophil subpopulations within tissue niches and environments. Neutrophils can prevail for up to 5 days and acquire inflammatory or resolving “memory” properties dependent upon local environments and tissue-derived signals [13, 14]. At the translational level, the establishment of potentially diverse “memory” neutrophil states is closely associated with human pathophysiological events such as acute sepsis as well as chronic diseases, including cancer and cardiovascular diseases [15–18]. With a particular note, the expansion of CD177^hi^ neutrophils has been implicated in the generation of innate exhaustion memory and associated with increased mortality in COVID-19 patients [19]. However, neutrophil reprogramming related to resolving inflammation is not well defined.

Sodium 4-phenylbutyrate (4-PBA) is an aromatic fatty acid analog with therapeutic potential in metabolic disorders [20–22], neurodegenerative diseases [23, 24], malignant illness [25], and inflammatory disorders [26, 27]. 4-PBA exerts anti-inflammatory properties by downregulating NF-κB activities and reducing endoplasmic reticulum (ER) stress [28–31]. In addition, 4-PBA may alleviate peroxisomal stress and dysfunction by binding to peroxisome proliferator-activated receptors (PPARs) [32–34]. The impaired peroxisomal homeostasis contributes to the generation of non-resolving inflammatory neutrophils related to the pathogenesis of chronic diseases [35, 36]. We previously reported that 4-PBA displays the immunomodulatory effect by restoring neutrophil peroxisome homeostasis and relieving chronic inflammation. Despite these intriguing phenotypic observations, a comprehensive characterization of 4-PBA-trained resolving neutrophils and underlying mechanisms are not currently available.

In the present study, we conducted single-cell RNA sequencing (scRNAseq) analyses of purified naive mature murine neutrophils as well as neutrophils trained by 4-PBA. We demonstrated that naive mature neutrophils were populated into three distinct subtypes based on their unique gene expression: exhausted, intermediate, and resolving. The resolving neutrophil population, in particular, was expanded upon 4-PBA stimulation with reduced pro-inflammatory mediator TNF-α production and increased homeostatic signatures, including CD200R, CD86, SERPINB1, and Resolvin D1. We validated our scRNAseq data with the independent characterization of key protein markers. Functionally, 4-PBA-programed neutrophils are more mature and exhibit enhanced phagocytic and bacterial-killing abilities. We also found that the PPARγ/LMO4/STAT3 signaling cascade is responsible for the generation of resolving neutrophils which are constitutively suppressed by TRAM, an adaptor protein in TLR4 signaling pathways associated with neutrophil exhaustion. Our findings reveal that neutrophils can potentially adopt distinct “memory” states and that resolving neutrophils can be expanded either through training with 4-PBA or genetic deletion of TRAM signaling adaptor.

## Results

### Generation of *Cd200r* expressing resolving neutrophils through 4-PBA

**Fig. 1:**
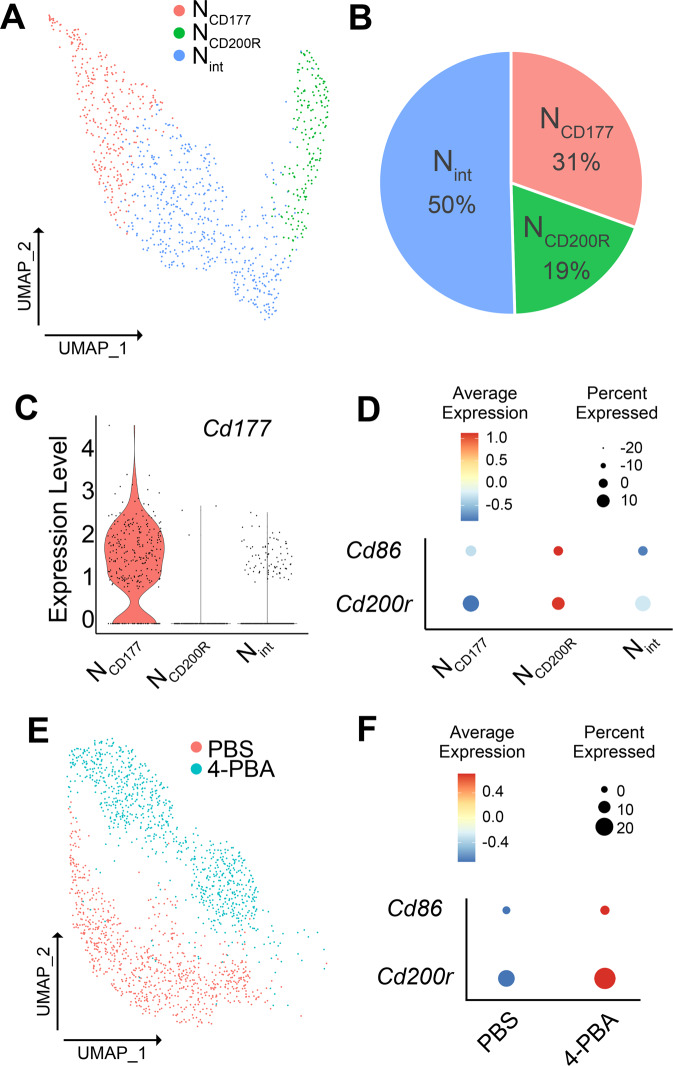
scRNAseq analysis of WT BM neutrophils. **A** The UMAP diagram of three subsets of neutrophils (exhausted N_CD177_; resolving N_CD200R_; and intermediate N_int_). **B** Relative percentages of three neutrophil subsets collected from naive WT mice bone marrow. **C** The violin plot of *Cd177* expression in three neutrophil subsets. **D** The bubble plot analysis of *Cd200r* and *Cd86* expression in three neutrophil subsets. **E** The UMAP diagram of neutrophils subjected to PBS or 4-PBA treatment. **F** The bubble plot analysis of *Cd200r* and *Cd86* levels in PBS or 4-PBA-treated neutrophils.

Emerging studies suggest that neutrophils can exert paradoxical and opposing effects ranging from exacerbating inflammation to facilitating homeostasis [1]. In order to better understand the heterogeneity of neutrophils, we conducted scRNAseq analysis of naive mature neutrophils purified from murine bone marrow (BM) based on Ly6G^+^ sorting. Consistent with several independent reports [37, 38], we observed that naive mature murine Ly6G^+^ neutrophils clustered into three distinct populations (Fig. 1A, B). We searched key identity genes within these three clusters by selecting functionally important genes involved in either neutrophil exhaustion (e.g., *Cd177*) or resolving characteristics (e.g., *Cd200r* and *Cd86*). Neutrophils with high CD177 expression have been associated with compromised immune functions and recently with COVID-19 disease severity and death [19]. On the other hand, higher levels of CD200R expression on neutrophils are associated with the enhanced anti-microbial function [39]. CD86 is a key co-stimulatory molecule expressed on neutrophils capable of enhancing adaptive immune functions [40]. Consistent with reports that reveal separate clusters of neutrophils with varying levels of CD177 [37, 41], we observed a cluster as CD177^hi^ cells (which we termed the N_CD177_ cluster), and the other two clusters were CD177^lo^ cells (Fig. 1C). The N_CD177_ cluster indeed had no expression of *Cd200r* nor *Cd86* (Fig. 1D), confirming previous observations that this cluster may represent the exhausted neutrophils with compromised immune functions. The other two clusters can be further separated based on the relative expression of *Cd200r* and *Cd86*, and we termed the other two CD177^lo^ populations as the minor population of N_CD200R_ (expressing higher levels of *Cd200r* and *Cd86*) and N_int_ (expressing an intermediate level of *Cd200r*) (Fig. 1D). Further analyses suggest that CD177 neutrophils not only exhibit pathogenic inflammatory features but also elevated expression of apoptotic genes (Supplementary Fig. S1A). The larger population of intermediate neutrophils also exhibits elevated expression of apoptotic genes (Supplementary Fig. S1B), consistent with previous findings of neutrophils with short-lived inflammatory phenotypes [37, 42, 43]. In contrast, the CD200R cluster not only preferentially expressed inflammatory resolving CD200R and immune-enhancing CD86 but also expressed elevated levels of anti-apoptotic genes (Supplementary Fig. S1C), suggesting a viable resolving phenotype with therapeutic potential. Similar clusters were observed through independent studies (Supplementary Fig. S1D) [37, 38].

We then treated purified naive mature murine neutrophils with either PBS or 4-PBA for 24 hours and collected cells for further scRNAseq analysis. As shown in Fig. 1E, 4-PBA reprogrammed all neutrophils into *Cd200r* and *Cd86*-expressing cells compared to control neutrophils (Fig. 1E, F). Our data reveal that neutrophils can be successfully reprogrammed into the *Cd200r* expressing population with potential resolving natures through in vitro training with 4-PBA.

### 4-PBA trained neutrophils potently express pro-resolving mediators

**Fig. 2:**
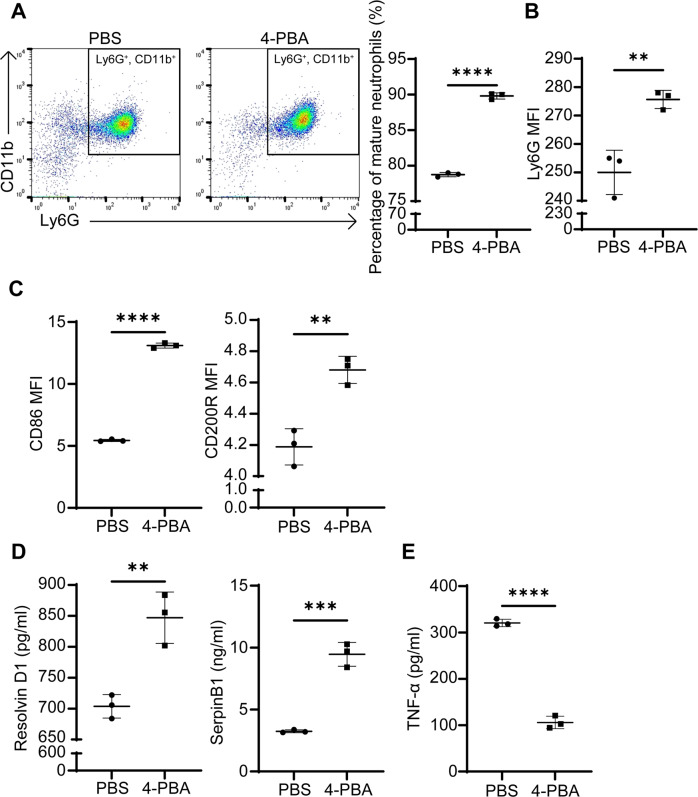
Resolving neutrophils trained by 4-PBA express elevated levels of cell surface CD86/CD200R, increased RvD1/SerpinB1, and reduced TNF-α. **A** Flow analyses of mature Ly6G^hi^CD11b^+^ neutrophils. WT neutrophils treated with PBS or 4-PBA (1 mM) for 24 hours were subjected to flow analyses and the percentages of Ly6G^hi^CD11b^+^ neutrophils were plotted. (*n* = 3). **B** The mean fluorescent intensity (MFI) levels of Ly6G on WT neutrophils treated with PBS or 4-PBA (1 mM) for 24 hours (*n* = 3). **C** The levels of CD86 and CD200R on WT neutrophils treated with PBS or 4-PBA (1 mM) for 24 hours (*n* = 3). **D** The expression of RvD1 and SerpinB1 from WT neutrophils treated with PBS or 4-PBA (1 mM) for 24 hours (*n* = 3). **E** The secretion of TNF**-**α from WT neutrophils treated with PBS or 4-PBA (1 mM) for 24 hours followed by PMA priming (100 ng/ml) for 30 min (*n* = 3). Data plotted as mean ± SD. ****P < 0.0001, ***P < 0.001, **P < 0.01 using two-sided Student’s *t* test.

Based on the intriguing clue from our scRNAseq data, we conducted independent confirmation studies through flow cytometry and ELISA approaches. Since 4-PBA reprogrammed all neutrophils into the *Cd200r* expressing population, we next conducted analyses comparing whole neutrophil populations treated with PBS or 4-PBA. We found that 4-PBA further enhanced neutrophil maturation with elevated Ly6G expression (Fig. 2A, B). This is consistent with our GO analysis demonstrating the differentially regulated genes in the categories of cell differentiation/maturation and neutrophil granulation in 4-PBA vs PBS-treated neutrophils (Supplementary Fig. S2). Next, we observed that neutrophils treated with 4-PBA expressed significantly higher levels of CD86 and CD200R proteins as compared to the PBS control group (~3-fold and ~1.15-fold induction, respectively) (Fig. 2C). In addition to cell membrane-associated resolving molecules, neutrophils are also potent secreting cells releasing soluble pro-resolving mediators such as SerpinB1 and lipid Resolvin D1 (RvD1). We, therefore, measured secreted levels of RvD1 and SerpinB1 through ELISA. As shown in Fig. 2D, we observed a nearly 100% increase of SerpinB1 and RvD1 in supernatants collected from neutrophils treated with 4-PBA as compared to PBS-treated control counterparts. SerpinB1 belongs to a superfamily of serine-protease inhibitors that can suppress the activities of inflammatory enzymes stored in neutrophil azurophilic granules to avoid unwanted cellular damage [9]. RvD1, a long-chain polyunsaturated fatty acid compound secreted by various immune cells, exerts the pro-resolving activity by reducing pro-inflammatory cytokine expression [4].

Our GO analysis of scRNAseq data also revealed genes in 4-PBA trained neutrophils involved in the negative regulation of cytokine production (Supplementary Fig. S2). We further examined the secretion potential of pro-inflammatory cytokine TNFα in treated neutrophils through ELISA. We observed that 4-PBA reduced the PMA-primed induction of TNF-α secretion in neutrophils by ~3-fold as compared to PMA-primed control neutrophils (Fig. 2E). Taken together, our data confirm that resolving signatures of 4-PBA treated neutrophils can be reflected at the protein levels with the elevated expression of immuno-enhancing (CD86) as well as anti-inflammatory and anti-microbial (CD200R, RvD1, SerpinB1) mediators and the decreased secretion of pro-inflammatory cytokine (TNF- α).

### The enhanced anti-microbial function of resolving neutrophils programmed by 4-PBA

**Fig. 3:**
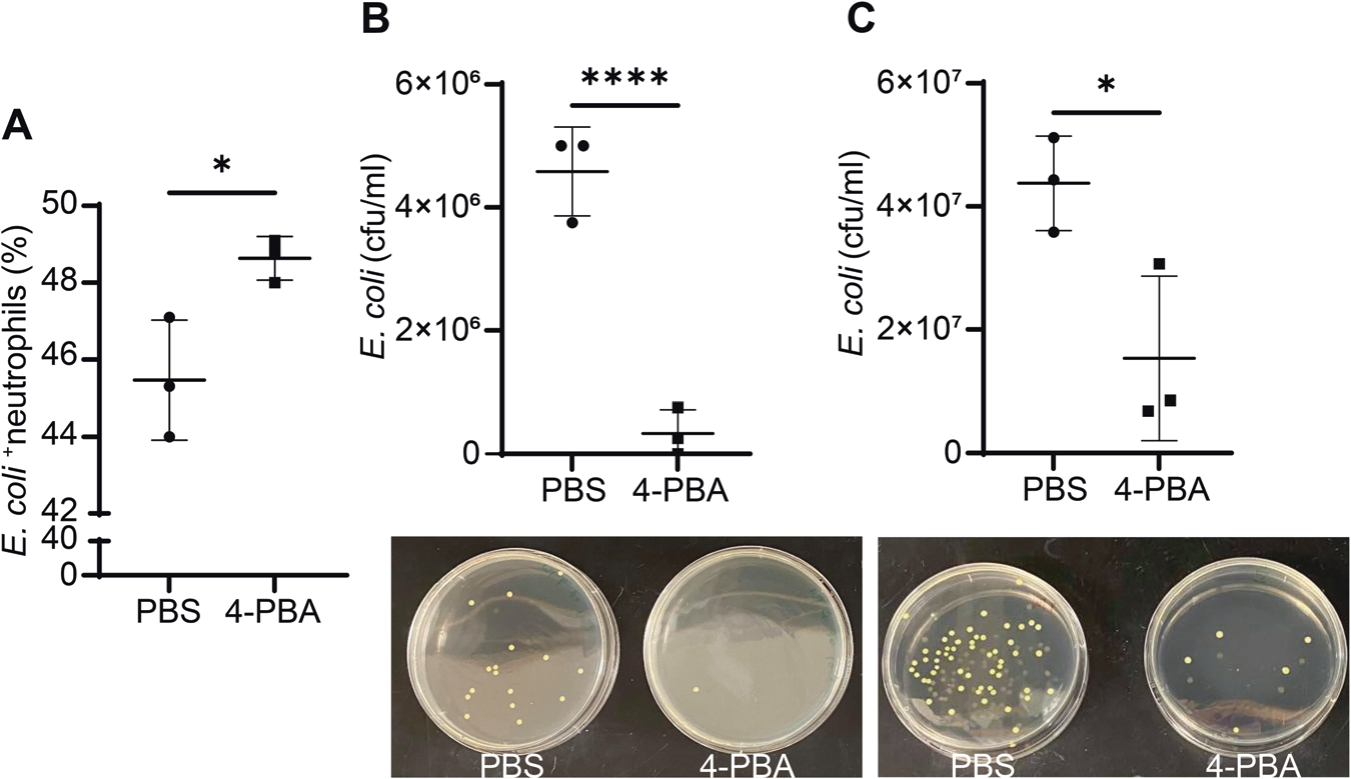
Resolving neutrophils trained by 4-PBA exhibit enhanced phagocytosis and bacterial-killing activities. **A** Flow analyses of neutrophil phagocytosis of GFP-labeled *E. coli*. WT neutrophils programmed with PBS or 4-PBA (1 mM; 24 hours) were subsequently co-incubated with GFP-labeled *E. coli* for 20 min. Following washing, neutrophils were subjected to flow analyses, and the percentages of GFP-*E. coli* containing neutrophils were counted and plotted (*n* = 3). **B** Analyses of bacterial killing through plating of viable *E. coli* harvested from lysed neutrophils. WT neutrophils programmed with PBS or 4-PBA (1 mM; 24 hours) were subsequently co-incubated with GFP-labeled *E. coli* for 30 min. Following washing, neutrophils were lysed and plated on bacterial culture plates. The numbers of viable E. coli were counted (bottom panel) and the CFU (colony-forming units) were plotted (upper panel) (*n* = 3). The representative image of viable intracellular *E. coli* from each group was shown. **C** Analyses of bacterial killing through plating of viable *E. coli* collected from culture supernatants. WT neutrophils programmed with PBS or 4-PBA (1 mM; 24 hours) were subsequently co-incubated with GFP-labeled *E. coli* for 30 min. Culture supernatants containing extracellular viable bacteria were plated (bottom panels showing representative culture images) and counted (upper panel) (*n* = 3). Data were plotted as mean ± SD. *P < 0.05 using two-sided Student’s *t* test. (**A**, **B**, and **C**).

CD200R is not only involved in resolving inflammation through suppressing inflammatory signaling processes but also involved in enhancing the anti-microbial functions of neutrophils [44, 45]. Given that 4-PBA can induce CD200R expression on neutrophils, we hypothesized that 4-PBA treatment may augment neutrophils’ anti-bacterial capacity. To test this, we performed neutrophil phagocytosis and bacterial-killing assays via flow cytometry as well as plating counting assay for the colony-forming unit (CFU), respectively. We found that neutrophils trained by 4-PBA exhibited enhanced phagocytosis of GFP-labeled *Escherichia coli* (*E. coli*) as compared to control neutrophils (Fig. 3A), suggesting that resolving neutrophils are more effective in removing microbes. We further examined the numbers of intracellular and extracellular viable bacteria after the co-incubation for 30 min. We observed that neutrophils trained by 4-PBA exhibited significantly elevated bacterial-killing capability, with almost no remaining viable *E. coli* inside the cells as compared to the control group in which there were around 4.5 million non-killed bacteria per 1 ml of lysed cell solution remaining intracellularly (Fig. 3B). Similarly, the numbers of viable extracellular bacterial colonies from the culture supernatants of 4-PBA-trained neutrophils were only a half of their control counterparts (Fig. 3C), showing the enhanced bacterial engulfing and killing activities of resolving neutrophils trained by 4-PBA. Collectively, these data demonstrate that 4-PBA can promote bacterial clearance ability to resolve neutrophils.

### 4-PBA reprograms resolving neutrophils through enhancing the signaling circuit involving PPARγ/LMO4/STAT3

**Fig. 4:**
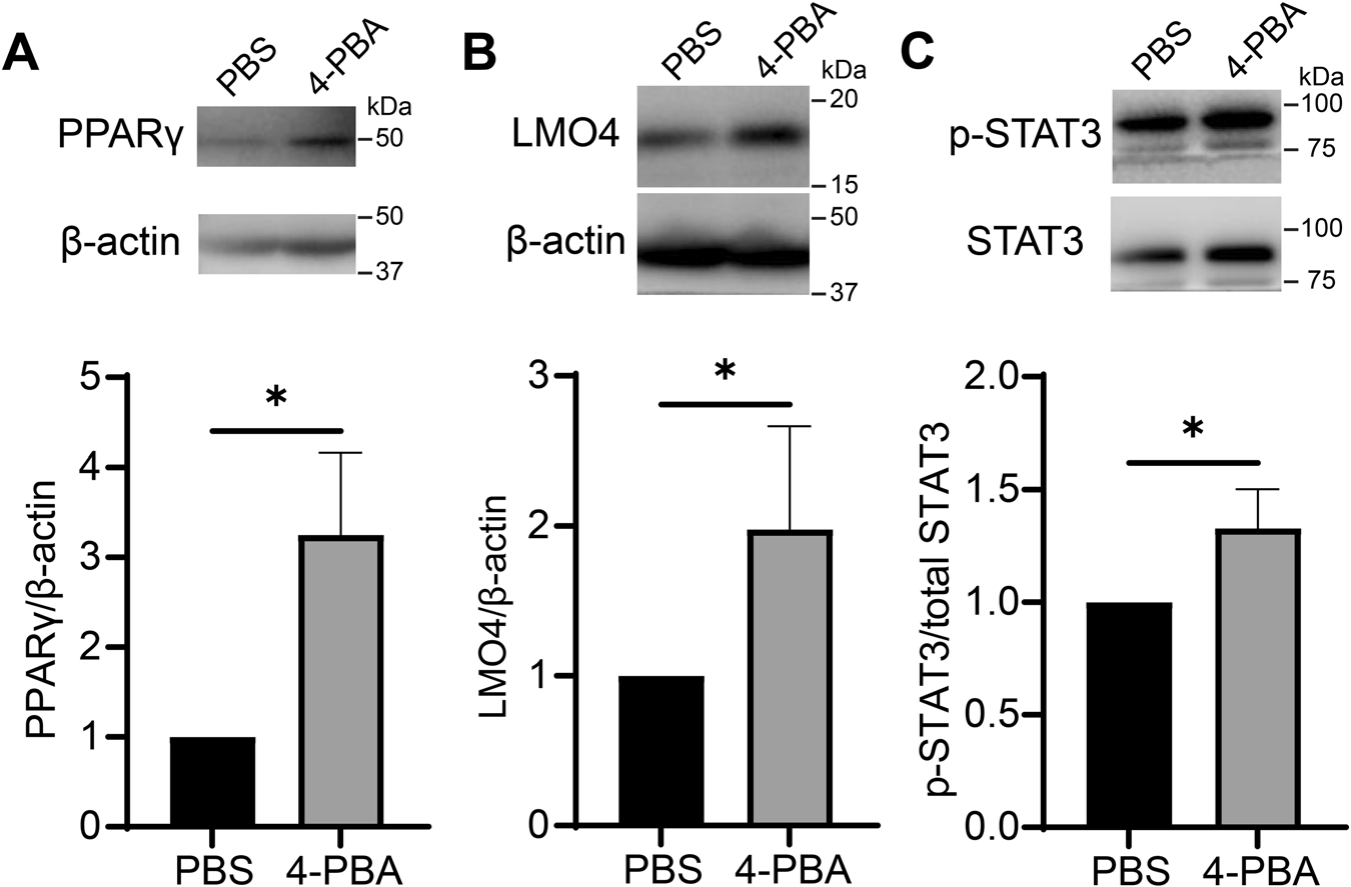
Induction of resolving signaling molecules PPARγ, LMO4, and p-STAT3 in neutrophils by 4-PBA. **A**–**C** The levels of PPARγ, LMO4, β-actin, p-STAT3, and total STAT3 of WT neutrophils treated with PBS or 4-PBA (1 mM; 24 hours) were determined by Western blot. Data were representative of at least three independent experiments and are plotted as mean ± SD. *P < 0.05 using Student’s *t* test.

We next examined molecular mechanisms potentially responsible for the generation of resolving neutrophils. In this regard, we evaluated the protein level of PPARγ, a transcription factor known to be involved in the expression of CD200R [46], as well as a key mediator for peroxisome biogenesis and homeostasis [47]. 4-PBA was shown to potently induce peroxisome homeostasis [36]. As expected, resolving neutrophils trained by 4-PBA had three times more PPARγ expression relative to the control ones (Fig. 4A, Supplementary Fig. S7A). Since the nuclear translocation of PPARγ is crucial to controlling the downstream transcription, we further examined the nuclear levels of PPARγ. Indeed, we observed that neutrophils treated with 4-PBA exhibited significantly higher levels of nuclear PPARγ as compared to control neutrophils (Supplementary Fig. S3, Supplementary Fig. S7C).

Moreover, the level of LMO4, a co-factor for PPARγ activation, was doubled in resolving neutrophils as compared to control neutrophils (Fig. 4B; Supplementary Fig. S7A). This finding complements a previous study reporting that LMO4 binds to PPARγ and augments PPARγ-dependent gene activation [48]. We further examined the expression of STAT3, which is one of the downstream effectors of PPARγ/LMO4 signaling [49]. LMO4 is critical for STAT3 activation and that activated STAT3 can further strengthen LMO4 signaling to form a positive feedback loop [50]. Meanwhile, STAT3 is an inhibitor of STAT1 which is a crucial driver for the generation of exhausted neutrophil phenotype reported by our group [51, 52]. We observed that 4-PBA significantly elevated phosphorylated STAT3 (Tyr705) levels in neutrophils by 50% as compared to the control neutrophils (Fig. 4C; Supplementary Fig. S7A). Together, our data reveal that resolving neutrophils exhibit increased levels of PPARγ, LMO4, and STAT3, reinforcing the hypothesis that PPARγ may form a positive loop with LMO4/STAT3 in the generation of resolving neutrophils [53].

### PPARγ and STAT3 are causally responsible for mediating 4-PBA reprogramming of resolving neutrophils

**Fig. 5:**
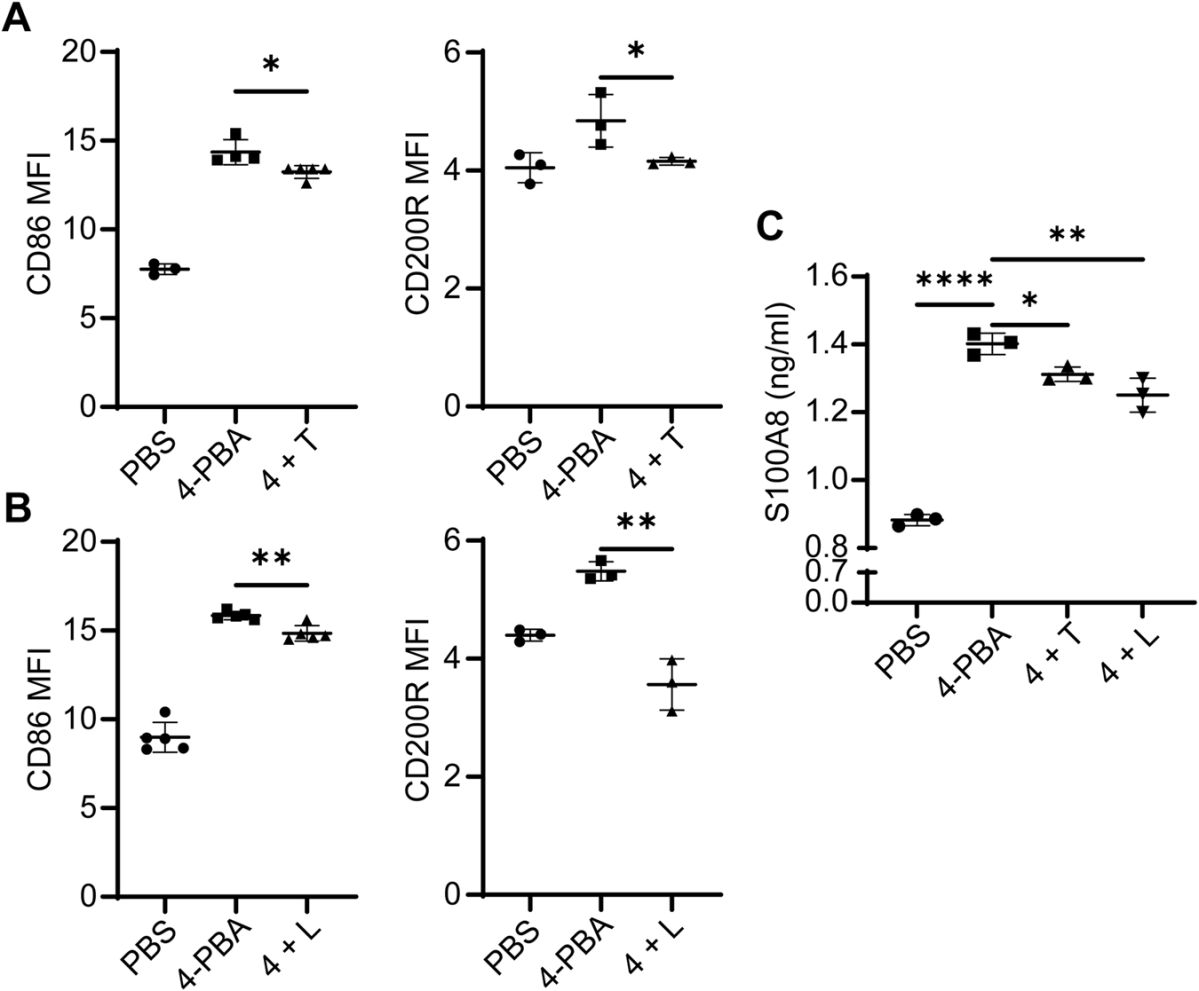
Inhibition of PPARγ or STAT3 reduces the generation of resolving neutrophils by 4-PBA. **A** Flow analyses of CD200R and CD86 levels on WT neutrophils treated with PBS, 4-PBA (1 mM; 24 hours) or pre-treated with T0070907 (0.5 µM) for 2 hours followed by 4-PBA (1 mM) stimulation for 24 hours (*n* = 3). **B** Flow analyses of CD200R and CD86 expression levels on WT neutrophils treated with PBS, 4-PBA (1 mM; 24 hours) or pre-treated with LLL12 (0.5 µM) for 2 hours followed by 4-PBA (1 mM) stimulation for 24 hours (*n* = 3). **C** ELISA analyses of S100A8 levels from WT neutrophils treated with PBS, 4-PBA (1 mM; 24 hours) or pre-treated with T0070907 (0.5 µM) or LLL12 (0.5 µM) for 2 hours followed by 4-PBA (1 mM) stimulation for 24 hours (*n* = 3). Data were plotted as mean ± SD. ** *P* < 0.01, * *P* < 0.05 using two-sided Student’s *t* test (**A** and **B**). ****P < 0.0001, ***P < 0.001, **P < 0.01, *P < 0.05 using one-way ANOVA test followed by the post-hoc Sidak multiple comparisons test (**C**). 4, 4-PBA; T, T0070907; L, LLL12.

To further test the causal contribution of PPARγ and STAT3 during the development of resolving neutrophils, we applied selective PPARγ or STAT3 inhibitor, T0070907 and LLL12, respectively, to the cell culture. We observed that neutrophils pre-treated with 0.5 µM T0070907 followed by the subsequent 4-PBA stimulation for 24 hours expressed less PPARγ as compared to control neutrophils with 4-PBA treatment only (Supplementary Figs. S4A and S7D). Likewise, neutrophils with LLL12 pretreatment together with 4-PBA stimulation exhibited less STAT3 activation as compared to 4-PBA treatment alone (Supplementary Figs. S4B and S7D). We then examined the relative expression of resolving mediators CD200R and CD86 in cells pre-treated with these inhibitors. We observed that the induction of CD86 and CD200R by 4-PBA was significantly blunted in neutrophils pre-treated with T0070907 as compared to control neutrophils treated with 4-PBA alone (Fig. 5A). Likewise, LLL12 also suppressed the elevation of CD86 and CD200R in neutrophils induced by 4-PBA (Fig. 5B).

Next, to elucidate the consequence of blocking PPARγ and STAT3 in mediating neutrophil microbicidal functions, we tested the levels of S100A8 which is an impotent anti-microbial granule component secreted by neutrophils [54]. Consistent with scRNAseq analysis showing enhanced maturation in 4-PBA programmed neutrophils (Supplementary Fig. S2), we observed elevated levels of S100A8 in 4-PBA trained neutrophils through ELISA assays (Fig. 5C). The application of the PPARγ inhibitor T0070907 or the STAT3 activation inhibitor LLL12 led to a significant reduction of S100A8 in neutrophils induced by 4-PBA (Fig. 5C). At the functional level, we further confirmed that the application of either PPARγ inhibitor T0070907 or the STAT3 activation inhibitor LLL12 significantly compromised bacterial-killing abilities to resolve neutrophils programmed by 4-PBA (Supplementary Fig. S5). Together, our data suggest that PPARγ and/or STAT3, at least partially, are causally responsible for the generation of resolving neutrophils.

### Genetic deletion of TRAM renders stable reprogramming of resolving neutrophils

We further evaluated the effects of TRAM deletion on the generation of resolving neutrophils. We previously reported that TRAM, a unique signaling adaptor in innate leukocytes involved in driving the pro-inflammatory polarization of both monocytes [55] and neutrophils [52]. Given the mutually inhibitory effects of pro- and anti-inflammatory signaling processes [56], we then asked whether the genetic deletion of TRAM may not only reduce the inflammatory polarization of neutrophils [52] but also facilitate the generation of resolving neutrophils. We conducted scRNAseq analyses of TRAM knock-out (KO) neutrophils. UMAP analysis revealed that TRAM KO neutrophils primarily clustered into only two populations, with a minor N_CD177_ subset (23% of the total TRAM KO population) and a majority of the resolving N_CD200R_ subset (77% of the total KO population) (Supplementary Fig. S6A, B). The proportion of the exhausted N_CD177_ subset was reduced (23% of the total TRAM KO population versus 31% of the total wild-type (WT) population). Heatmap analysis further revealed that both populations were *Cd200r* positive (Supplementary Fig. S6C), suggesting the TRAM deletion renders the generation of resolving neutrophils.

**Fig. 6:**
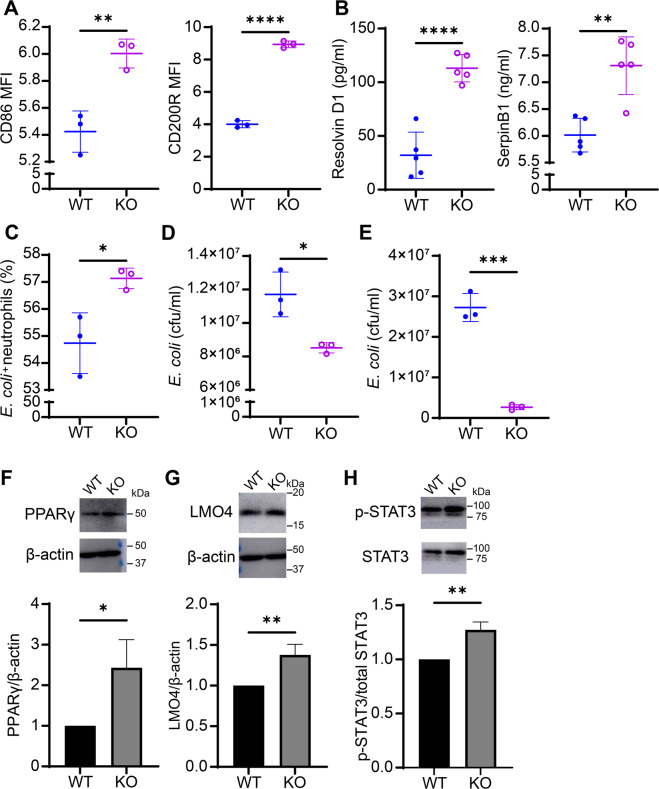
Genetic deletion of TRAM renders constitutive generation of resolving neutrophils. **A** Flow analyses of CD86 and CD200R expression levels on WT or TRAM KO neutrophils (*n* = 3). **B** The levels of Resolvin D1 and SerpinB1 from the supernatant of overnight-cultured WT or TRAM KO neutrophils (*n* = 3). **C** Flow analyses of neutrophil phagocytosis of GFP-labeled *E. coli*. Purified WT or TRAM KO neutrophils were co-incubated with GFP-labeled *E. coli* for 20 min. Following washing, neutrophils were subjected to flow analyses, and the percentages of GFP-*E. coli* containing neutrophils were counted and plotted (*n* = 3). **D** Analyses of bacterial killing through plating of viable *E. coli* harvested from lysed neutrophils. WT or TRAM KO neutrophils were co-incubated with GFP-labeled *E. coli* for 30 min. Following washing, neutrophils were lysed and plated on bacterial culture plates. The numbers of viable E. coli were counted and the CFU were plotted (*n* = 3). **E** Analyses of bacterial killing through plating of viable *E. coli* collected from culture supernatants. WT or TRAM KO neutrophils were co-incubated with GFP-labeled *E. coli* for 30 minutes. Culture supernatants containing extracellular viable bacteria were plated and counted with the CFU plotted (*n* = 3). **F**–**H** The levels of PPARγ, LMO4, β-actin, p-STAT3, total STAT3 of overnight-cultured WT or TRAM KO neutrophils were determined by Western blot. Data are representative of at least three independent experiments. Data plotted as mean ± SD. ****P < 0.0001, ***P < 0.001, **P < 0.01, *P < 0.05 using two-sided Student’s *t* test.

We then performed independent analyses measuring levels of key resolving proteins. Through flow cytometry analysis comparing overnight-cultured WT and TRAM KO neutrophils, we observed that TRAM KO neutrophils expressed significantly higher levels of CD86 and CD200R as compared to WT neutrophils (Fig. 6A). Furthermore, TRAM-deficient neutrophils secreted twice as much RvD1 and 125% higher level of SerpinB1 as compared to WT ones (Fig. 6B), suggesting that TRAM deletion can polarize neutrophils into a resolving state. We further corroborated our observation by assessing neutrophil anti-bacterial functions through phagocytosis and killing assays as described above. Indeed, TRAM-deficient neutrophils were more competent in engulfing and killing *E. coli*, manifested in less viable intracellular and extracellular bacteria remaining in the KO neutrophils (1.3 times and 10 times less, respectively), indicating that TRAM restrains neutrophil microbicidal functions (Fig. 6C–E). Given our observation that 4-PBA can promote the generation of resolving neutrophils by sustaining the PPARγ/LMO4/STAT3 positive feedback loop and that TRAM-deficient neutrophils constitutively have resolving characteristics, we further tested whether TRAM deletion may affect the levels of those resolving-related transcription factors. We found that the protein expression of PPARγ (as well as nuclear PPARγ), LMO4, and phosphorylated STAT3 were all considerably elevated in TRAM KO neutrophils as compared to their WT counterparts (Fig. 6F–H; Supplementary Figs. S3, S7B, S7C). Our findings suggest that TRAM may block the generation of resolving-associated gene regulators and that removing TRAM may render sustained elevation of PPARγ/LMO4/STAT3 signaling circuitry enabling the generation of resolving neutrophils.

## Discussion

In this study, we showed that neutrophils can be effectively reprogrammed through either pharmacological treatment with 4-PBA or genetic deletion of TRAM into a resolving state with overall elevated expression of CD200R, RvD1; reduced expression of pro-inflammatory mediator TNFα; and enhanced bactericidal capacity. Mechanistically, our data revealed that the generation of resolving neutrophils is mediated by the PPARγ/LMO4/STAT3 signaling circuitry which can be induced by 4-PBA and is constitutively suppressed by TRAM.

Previous studies suggest that both resting murine and human neutrophils tend to exhibit immune suppressor functions that impair T cell survival and function [57–59]. Our scRNAseq analyses reveal that the majority of the WT mature neutrophils do not express the homeostatic CD200R or CD86. Our data are consistent with emerging scRNAseq analyses of mature neutrophils generally clustering into populations with immune suppressive/exhausted functions [37]. Indeed, the two majority subsets of neutrophils are CD177^hi^ exhausted neutrophils and intermediate neutrophils, both with no expression of CD200R nor CD86. In addition to being inflammatory, both CD177 and the intermediate cluster exhibit higher levels of apoptotic genes, consistent with the observed short-lived apoptotic phenotype [37, 38]. In contrast, our data reveal the presence of a small resting neutrophil subset with a higher CD200R and CD86-expressing signature, potentially representative of resolving neutrophils, and reconcile opposing phenotypes assigned to neutrophils in previous literature. The CD200R cluster also expresses higher levels of selected anti-apoptotic genes such as BCL10 and MCL1.

With a clear translational implication, our current study identifies effective approaches in expanding the population of the CD200R/CD86 expressing resolving neutrophil population by 4-PBA. Notably, we showed that all subsets of resting neutrophils are converted into CD200R/CD86 expressing resolving neutrophils upon treatment by 4-PBA. This finding justifies our current comparative analyses of the whole neutrophil populations without or with 4-PBA treatment. We also demonstrated that 4-PBA can further enhance the extent of neutrophil maturation as reflected in elevated cell surface Ly6G levels and increased levels of S100A8. Interestingly, 4-PBA treatment not only induces a global reprogramming of matured neutrophils with resolving potentials but can also suppress the secretion of pro-inflammatory mediator TNFα. Similarly, our data reveal that genetic deletion of the inflammatory adaptor molecule TRAM can also facilitate the expansion of the CD200R/CD86 expressing resolving neutrophils.

Our data further reveal that expanded CD200R expressing neutrophils exhibit enhanced expression of both RvD1 and SerpinB1, potent mediators in resolving acute and chronic inflammation by enhancing efferocytosis of apoptotic cells, reducing endothelial inflammation, and improving vasculature integrity [5, 60–63]. In addition, our functional data validate that resolving neutrophils reprogrammed by 4-PBA or TRAM deletion exhibit enhanced phagocytosis and bacterial-killing activities with elevated anti-microbial granule secretion. S1008A is a potent anti-microbial agent released by neutrophils during the degranulation process [54]. Although S1008A was also viewed as inflammatory, emerging studies indicate that S1008A has anti-inflammatory effects through scavenging ROS, reducing oxidative damage, and promoting IL-10 expression [64], beneficial for the regression of cardiovascular diseases [65] and resolution of inflammation [66]. Altogether, neutrophils with resolving potentials may serve as novel vehicles to resolve inflammation without compromising host defense, holding significant promises in the future treatments of both acute (e.g., polymicrobial infections and sepsis) as well as chronic diseases (e.g., cardiovascular inflammation) [67, 68].

Emerging studies suggest that human neutrophils can also adopt dynamic functional characteristics during health and disease, sharing conserved features with murine neutrophils [37]. Increased CD177-expressing exhausted neutrophils are reported in several clinical conditions including sepsis [69, 70]. Recent studies suggest that upregulated expression of granulocyte *CD177* may serve as a potential marker for the physiopathology of COVID-19 in patients [19, 71]. Our data revealing increased apoptotic signatures among intermediate and CD177 neutrophils are consistent with previous phenotypic observations of neutrophils with generally short-lived and inflammatory behaviors [37, 42, 43]. Further comparative analyses of neutrophil subsets in mice and humans under healthy and diseased conditions are needed to better harness the therapeutic potential of resolving neutrophils. Our current study provides important clues and guidance for future targeted studies clarifying neutrophil dynamics in mice and humans related to health and disease.

Our study further provides a novel mechanism responsible for the expansion of resolving neutrophils that involve the coupled elevation of PPARγ, LMO4, and p-STAT3. As a potent PPARγ ligand, 4-PBA can activate PPARγ to relieve oxidative stress [36, 72]. Consistent with the previous findings, we showed that resolving neutrophils expanded by either 4-PBA treatment or TRAM deletion expressed significantly higher levels of PPARγ as compared to resting naive WT neutrophils and enable nuclear translocation of PPARγ to exert its immunomodulatory functions. Together, our data identify a coupled sustaining circuitry involving PPARγ/LMO4/STAT3 that can effectively aid the generation of resolving neutrophils.

Although we revealed selected features of resolving neutrophils, other relevant signatures of resolving neutrophils may not be properly captured through our current study. Post-transcriptional modifications of expressed proteins may also be highly important in modulating neutrophil functions, especially considering the rapid responsiveness of neutrophils upon stimulation. Likewise, besides TRAM, other adaptor molecules such as TRIF and MyD88 may also participate in the dynamic polarization of neutrophil subsets. Future systems characterization of resolving neutrophils will be needed to better harness the therapeutic potential of resolving neutrophils for the treatment of acute and chronic diseases including sepsis and cardiovascular diseases.

## Materials and methods

### Mice and bacteria

Both WT and TRAM-deficient mice on the C57BL/6 background, ages 6–10 weeks and weighing 20–30 grams, were purchased from the Jackson Laboratory and maintained under specific pathogen-free conditions with standard chow and water ad libitum at the animal facility of Virginia Tech in accordance with the Institute for Animal Care and Use Committee–approved protocol. *Escherichia coli* GFP that has the green fluorescence labeling was purchased from ATCC.

### In vitro neutrophil culture

BM neutrophils were collected from WT and TRAM-deficient mice and isolated on 62.5% Percoll gradient as we described previously [52]. Cells were then cultured in a complete RPMI medium (10% fetal bovine serum, 2 mM l-glutamine, and 1% penicillin/streptomycin) supplemented with 100 ng/ml G-CSF in the presence of PBS or 4-PBA (1 mM) (TOCRIS, no. 2682) for 24 hours. Neutrophils might be further treated with PMA (100 ng/ml) or LPS (10 µg/ml) for 30 min as stated. In p-STAT3 and PPARγ blockage experiments, purified neutrophils were pre-treated with 0.5 µM LLL12 (BioVision, no. 1792) or 0.5 µM T0070907 (Cayman Chemical, no. 10062) for 2 hours before being stimulated with 4-PBA unless otherwise indicated.

### Single-cell sequencing and analysis

BM neutrophils were harvested from WT C57BL/6 mice and TRAM-deficient mice cultured as indicated above with either PBS or 0.1 mM 4-PBA for 24 hours [52]. cDNA libraries were generated by using the 10X Genomics Chromium Single Cell 3’ Reagent Kits (version 3 Chemistry), and sequenced by Novogen on the Illumina® HiSeq platform as we reported [73] and briefly described below. Around 1000 single-cell gel beads in emulsion per sample were prepared. The cDNAs of each sample were amplified for 12 cycles, quantified by Qubit, quality checked by Bioanalyzer in order to verify the size distribution of cDNA samples (~400 bp), and used for library preparation. Indexed library samples following the quality control evaluation by Tapestation were quantified by the KAPA library quantification kit (Universal). Pooled library samples were sequenced by Novogen through pair-end sequencing on Illumina® HiSeq 4000 platform, with the read length of 150 bp at each end plus 8-bp i7 index. Raw sequencing data were analyzed using the Cell Ranger (version 3.1.0) with mouse reference genome and annotation (Cell Ranger reference version 3.3.0, mm10, Ensembl 93) from the 10X Genomics website (https://support.10xgenomics.com/single-cell-gene-expression/software). Cell Ranger (version 3.0.2) was used to perform the alignment and mapping of sequenced reads, as well as the quantification of relative levels of gene expression. Quality control and data normalization were performed using the default pipeline of Seurat (version 3.1.4) in R [74] which filtered out doublets. Cells that had fewer than 200 unique genes were excluded; genes that existed in fewer than three cells were also removed. Dimensionality reduction was performed by principal component analysis, and cells were clustered through UMAP analysis as we reported [73]. Gene enrichment GO analyses were performed as we described [75]. Relevant datasets were deposited at the NCBI database with accession # GSE182356.

### Flow cytometry

Cells obtained post-culture were harvested, blocked with Fc block (1:200 dilution; BD Biosciences, no. 553141), and stained with anti-Ly6G (1:200 dilution; BioLegend, no. 127606), anti-CD11b (1:200 dilution; BioLegend, no. 101226), anti-CD86 (1:200 dilution; BioLegend, no. 105006), and anti-CD200R antibodies (1:200 dilution; BioLegend, no. 123908). Annexin V/PI staining kit (1:4000 dilution; Thermo Fisher Scientific, no. P3566) was used to evaluate the cell viability. Antibody-labeled samples were analyzed with a FACSCanto II (BD Biosciences), and data were analyzed with FlowJo (Ashland, OR).

### ELISA

The supernatant of post-cultured cells as described above was collected. The levels of Resolvin D1 and SerpinB1 in samples were measured by the ELISA kits purchased from MyBioSource (no. MBS7256343 and MBS7245914, respectively). The levels of S100A8 in samples were accessed by the ELISA kits from Thermo Fisher (no. EM67RB).

### Neutrophil phagocytosis and bacterial-killing assays

Bacterial cultures were diluted to a concentration of 10^8^ CFU in 1 ml 10% normal mouse serum (NMS) in HBSS for 30 min for opsonization. Six × 10^5^ neutrophils were mixed with opsonized bacteria in the same media (multiplicity of infection = 1:10) and then incubated at 37 °C on a shaker rotating at 25 rpm. Samples were prepared in triplicate. For the phagocytosis assay, samples were immediately placed on ice after a 20-minute incubation. Each sample was centrifuged to pellet the neutrophils which were then stained with anti-Ly6G antibody and analyzed with the flow cytometry machine (as described above) to examine the percentage of neutrophils with intracellular bacteria. For the bacterial-killing assay, both viable bacteria inside the cells and in suspensions were evaluated. In short, after 30 min of incubation, samples were left on ice followed by being washed twice with ice-cold PBS at 100 × *g* for 5 min. Neutrophils were then lysed in 0.05% Triton X, and the viable intracellular bacteria (CFU) were calculated by plating on TSA (4% TSA/100 µg/ml Ampicillin). In different sets of experiments, the suspension of each post-incubated sample was directly applied to TSA plates after the serial dilution to determine the number of viable bacteria left in the suspensions. The plates were incubated overnight at 37 °C, and the colonies were counted manually.

### Western blot

Western blot was performed as previously described [55]. Briefly, cells were spun down and lysed in the 2% SDS lysis buffer containing protease and phosphatase inhibitor cocktail mixture (Sigma; no. P8340, P5726, and P0044). Cell lysates were incubated on ice for at least 20 min and sonicated for a couple of seconds. The protein concentration of each sample was determined using the Bio-Rad DC Protein Assay kit (Bio-Rad Laboratories; no. 5000112). For extracting nuclear and cytoplasmic samples, we used a commercial kit to collect cytosolic and nuclear fractions separately as the manufacturer’s protocol instructed (Thermo Fisher; no. 78833). Ten percent acrylamide gel was used to separate proteins by size which were then transferred to PVDF membranes. After being blocked with 5% non-fat milk for 1 hour, membranes were probed with targeted protein antibodies overnight at 4 °C. Lastly, HRP-linked anti-rabbit IgG or anti-mouse IgG Ab and the ECL detection kit (VWR; no. 490005-020) were used to detect the blots. ImageJ software (NIH) was used for the relative protein quantification by normalizing each target to its respective housekeeping protein or total protein expression and normalizing these values to the control (PBS) group subsequently.

### Statistical analysis

All experiments were repeated at least three times. Prism (GraphPad Software 9.0, La Jolla, CA) was used to generate graphs and determine statistical significance using Student’s *t* test. *p* < 0.05 was considered statistically significant.

## Supplementary information

Supplementary file clean copy

checklist

## Data Availability

All data needed to evaluate the conclusions are presented in the paper. Additional data related to this paper may be requested from the corresponding author.
