# Supplementary material for: Generation of resolving memory neutrophils through pharmacological training with 4-PBA or genetic deletion of TRAM
Source: Cell Death Dis. 2022 Apr 13;13(4):345. doi: 10.1038/s41419-022-04809-6 (PMC9007399; doi:10.1038/s41419-022-04809-6)
Supplement: Supplementary file 1 — Supplementary file clean copy [file 41419_2022_4809_MOESM1_ESM.pdf]

Supplemental Figures

Generation of resolving memory neutrophils through pharmacological training with 4-PBA or genetic deletion of TRAM

RuiCi Lin[1], Ziyue Yi[1,2], Jing Wang[1], Shuo Geng[1], Liwu Li[1, 2]

[1]Department of Biological Sciences, Virginia Tech, Blacksburg, VA24061; [2]Graduate Program of Genetics, Biotechnology and Computational Biology, Virginia Tech, Blacksburg, VA24061

Running Title: Generation of resolving neutrophil memory

Keywords: Neutrophil memory, Inflammation resolution, CD200R, TRAM, 4-PBA

*Correspondence:

Liwu Li, Ph.D

970 Washington Street, Virginia Tech, Blacksburg, VA 24061-0910 Email: lwli@vt.edu

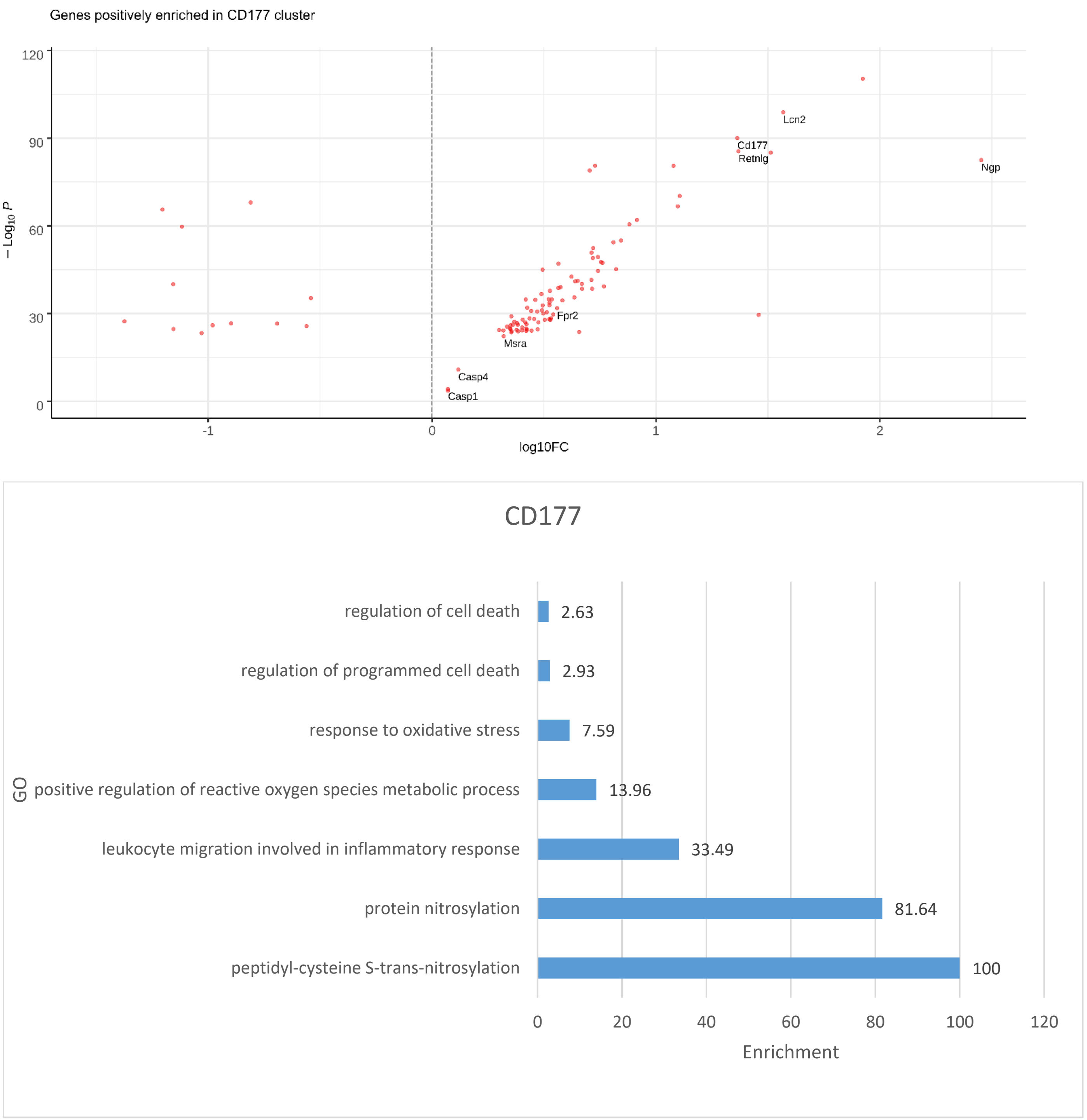

Supplementary Figure S1A. Differential gene expression profiles in the CD177 neutrophil cluster. ***Upper panel***: Volcano plot demonstrating up-regulated genes in the CD177 neutrophil cluster (fold changes in x axis; Mann-Whitney p value in y axis). ***Lower panel***: Enrichment analyses of GO biological process categories of significantly upregulated genes in CD177 neutrophil cluster.

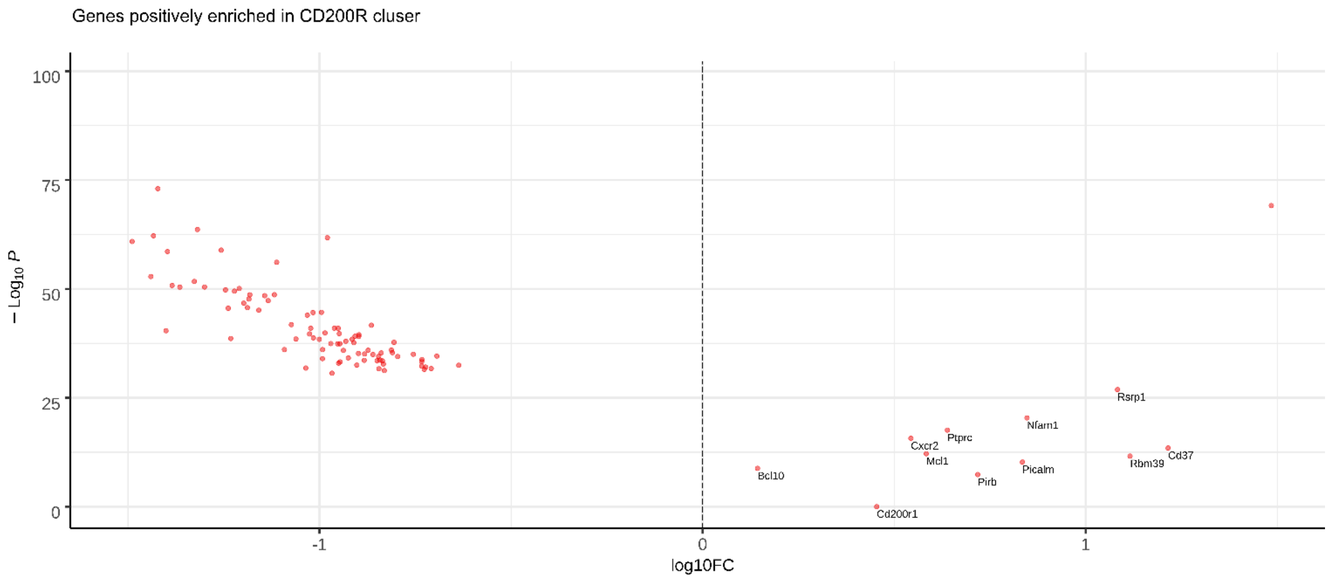

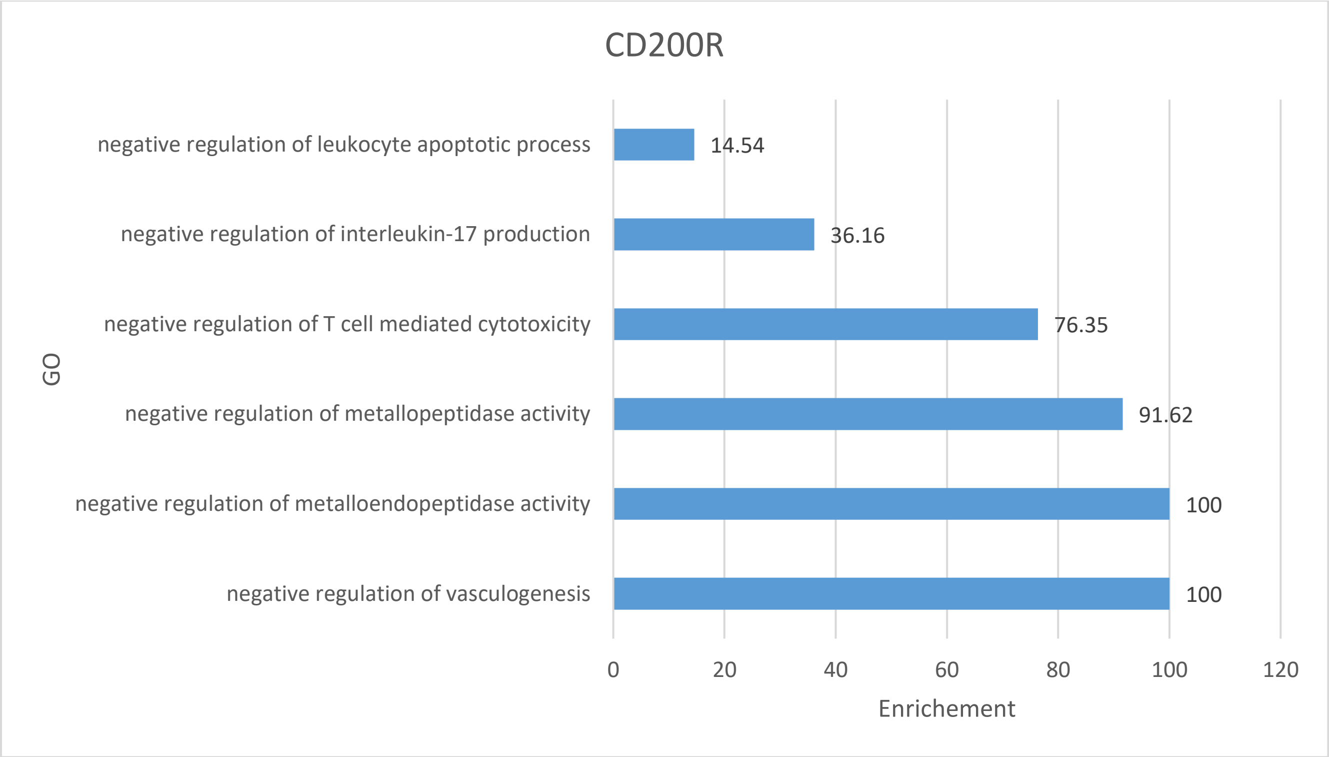

Supplementary Figure S1B. Differential gene expression profiles in the CD200R neutrophil cluster. ***Upper panel***: Volcano plot demonstrating up-regulated genes in the CD200R neutrophil cluster (fold changes in x axis; Mann-Whitney p value in y axis). ***Lower panel***: Enrichment analyses of GO biological process categories of significantly upregulated genes in CD200R neutrophil cluster.

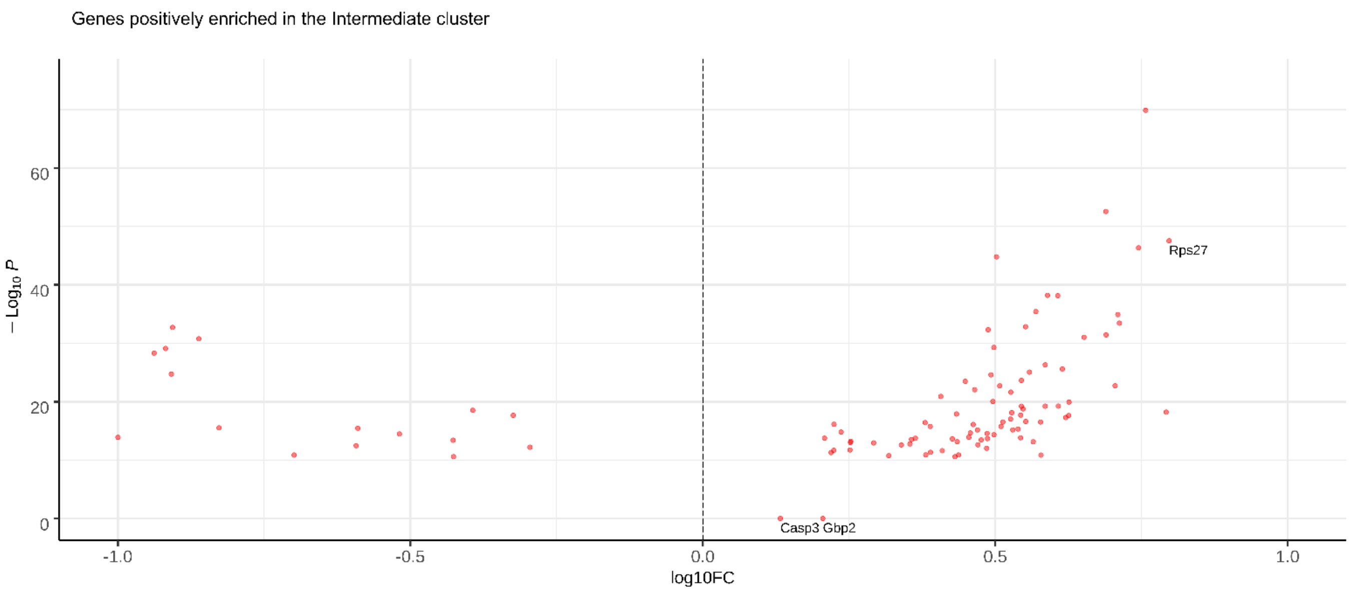

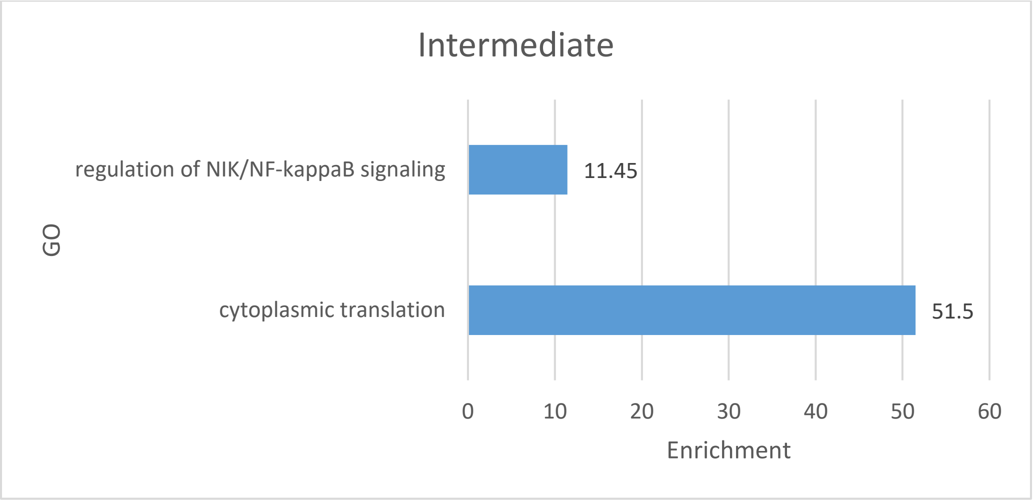

Supplementary Figure S1C. Differential gene expression profiles in the intermediate neutrophil cluster. ***Upper panel***: Volcano plot demonstrating up-regulated genes in the intermediate neutrophil cluster (fold changes in x axis; Mann-Whitney p value in y axis). ***Lower panel***: Enrichment analyses of GO biological process categories of significantly upregulated genes in intermediate neutrophil cluster.

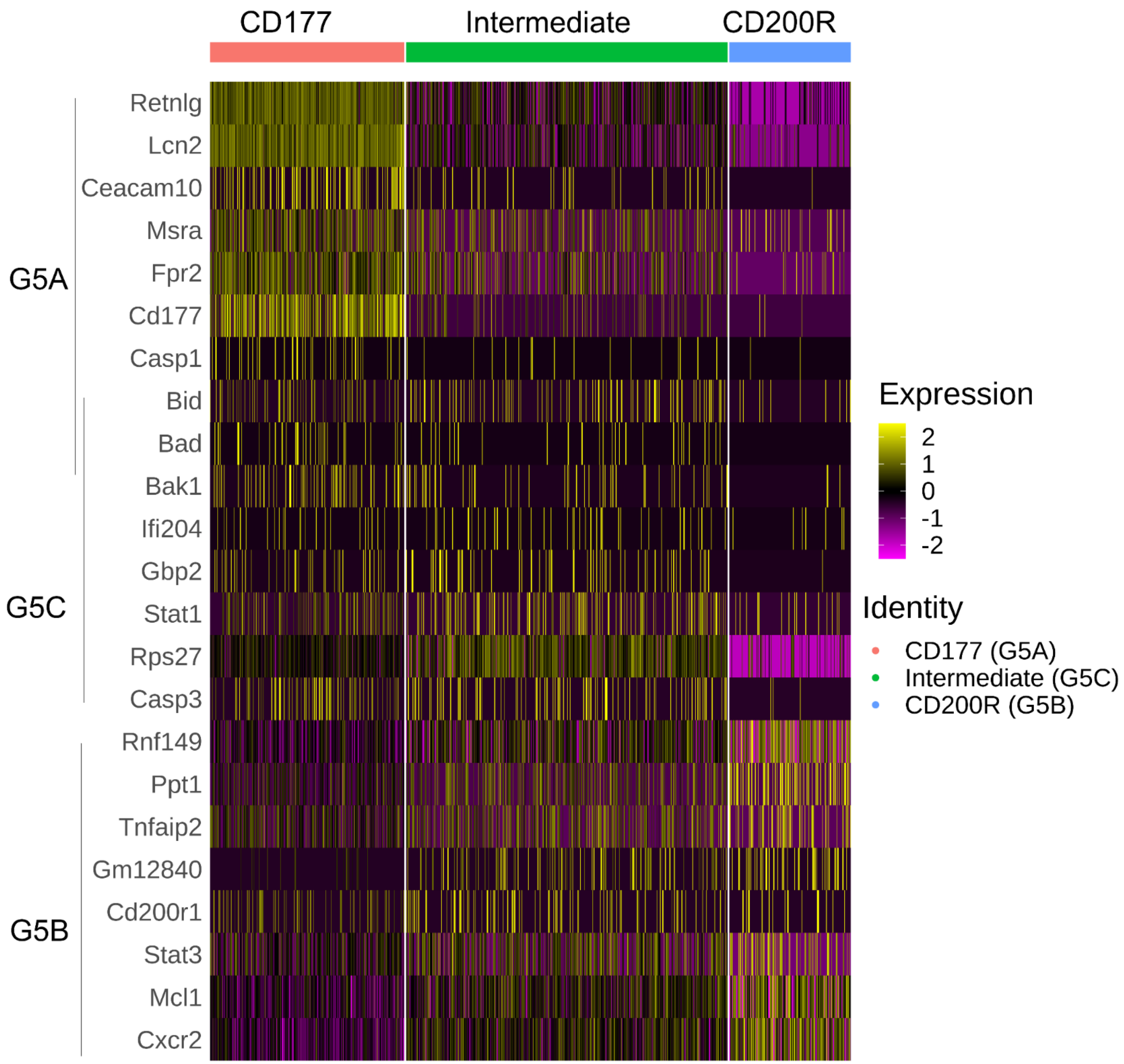

Supplementary Figure S1D. Comparative analyses of three neutrophil clusters. Signature genes enriched in three neutrophil clusters (5a, 5b, 5c) by an independent study (Xie et al, 2020, Nature Immunology, 21:1119-1133) correlated with the three clusters defined in this report, with the 5a cluster representing the CD177 population; 5c cluster correlating with the intermediate population; 5b relating to CD200R population.

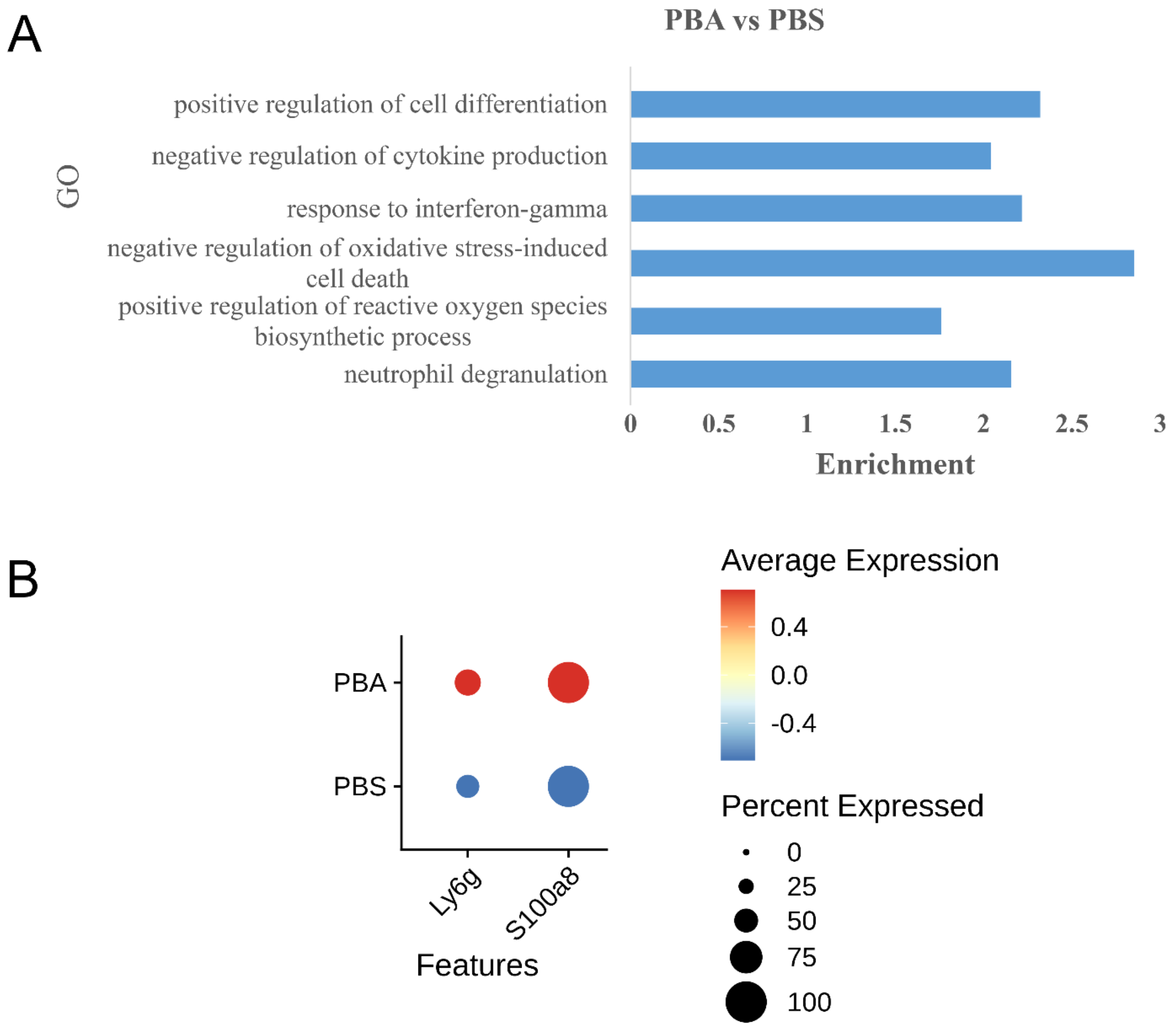

Supplementary Figure S2. GO analyses of scRNAseq data from 4-PBA vs PBS programmed neutrophils.

A Enrichment analyses of GO biological process categories representing significantly altered genes comparing 4-PBA vs PBS trained neutrophils.

B Bubble plot analyses of key neutrophil maturation genes Ly6G and S100A8 comparing PBS and 4-PBA trained neutrophils.

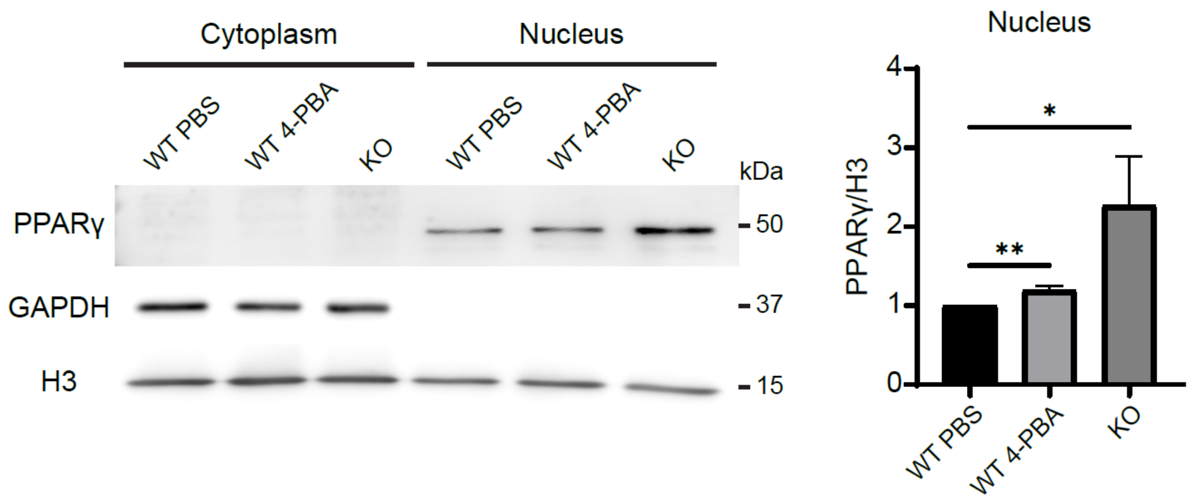

Supplementary Figure S3. Nuclear and cytoplasmic levels of PPARγ.

(Left panel): Western blot analyses of PPARγ, GAPDH, and H3 levels from the cytosolic or nuclear fractions of neutrophils treated as specified. (Right panel): Quantification data from three experiments. Data were plotted as mean ± SD. **P<0.01, *P<0.05 using one-way ANOVA test followed by the post-hoc Sidak multiple comparisons test.

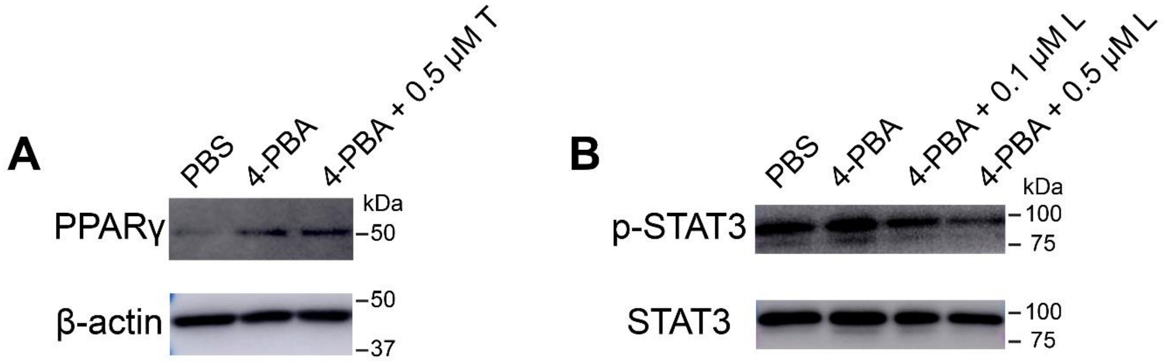

Supplementary Fig. S4 Inhibition of PPARγ or STAT3 by selective inhibitors.

A Western blot of PPARγ and β-actin on WT neutrophils treated with PBS, 4-PBA (1 mM; 24 hours), or pre-treated with T0070907 (0.5 μM) for 2 hours followed by 4-PBA (1 mM) stimulation for 24 hours.

B Western blot of p-STAT3 and STAT3 of WT neutrophils treated with PBS, 4-PBA (1 mM; 24 hours), or pre-treated with LLL12 (0.1 or 0.5 μM) for 2 hours followed by 4-PBA (1 mM) stimulation for 24 hours.

Data are representative of at least three independent experiments. 4, 4-PBA; T, T0070907; L, LLL12.

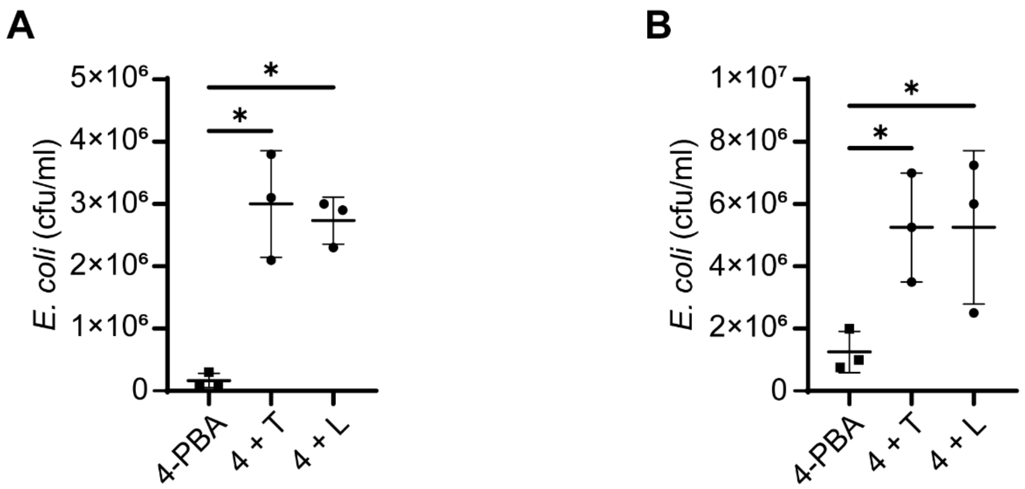

Supplementary Fig. S5 Inhibition of PPARγ or STAT3 reduces bacterial killing abilities of resolving neutrophils trained by 4-PBA.

A Analyses of bacterial killing through plating of viable *E. coli* harvested from lysed neutrophils. WT neutrophils pre-treated without or with T0070907 (0.5 µM) or LLL12 (0.5 µM) for 2 hours followed by 4-PBA (1 mM) stimulation for 24 hours. Neutrophils were subsequently co-incubated with GFP-labeled *E. coli* for 30 minutes. Following washing, neutrophils were lysed and plated on bacterial culture plates. The numbers of viable *E. coli* were counted and the CFU (colony forming units) were plotted (n = 3).

B Analyses of bacterial killing through plating of viable *E. coli* collected from culture supernatants. WT neutrophils pre-treated without or with T0070907 (0.5 µM) or LLL12 (0.5 µM) for 2 hours followed by 4-PBA (1 mM) stimulation for 24 hours. Neutrophils were subsequently co-incubated with GFP-labeled *E. coli* for 30 minutes. Culture supernatants containing extracellular viable bacteria were plated and counted (n = 3). Data were plotted as mean ± SD. *P<0.05 using one-way ANOVA test followed by the post-hoc Sidak multiple comparisons test. 4, 4-PBA; T, T0070907; L, LLL12.

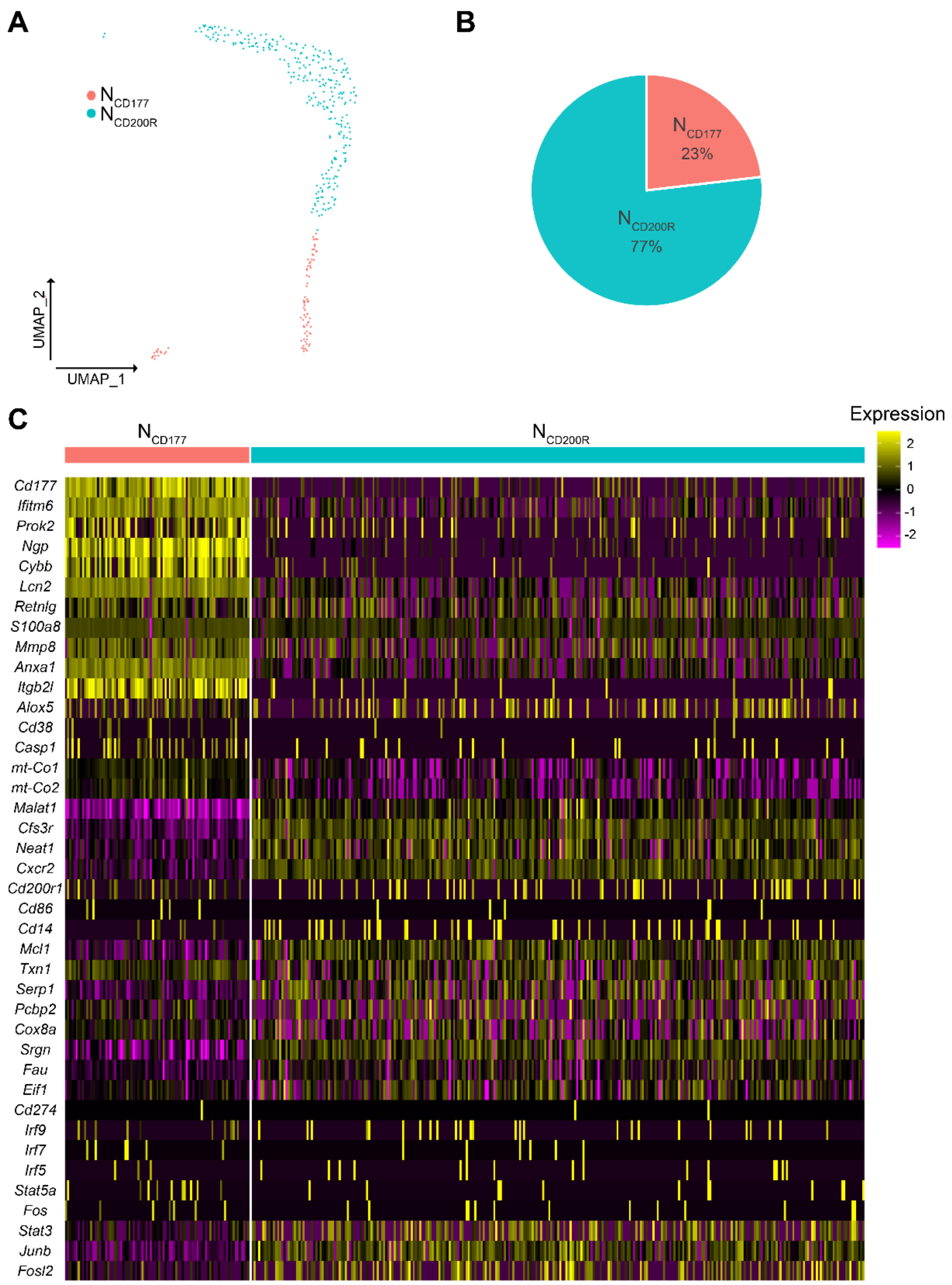

Supplementary Figure S6. scRNAseq analysis of TRAM-deficient BM neutrophils.

A The UMAP diagram of two TRAM-deficient BM neutrophil subsets (N_{CD177} and N_{CD200R}).

B Relative percentages of two neutrophil subsets collected from TRAM-deficient mice bone marrow.

C The heatmap of representative genes enriched in two subsets of TRAM-deficient BM neutrophils.

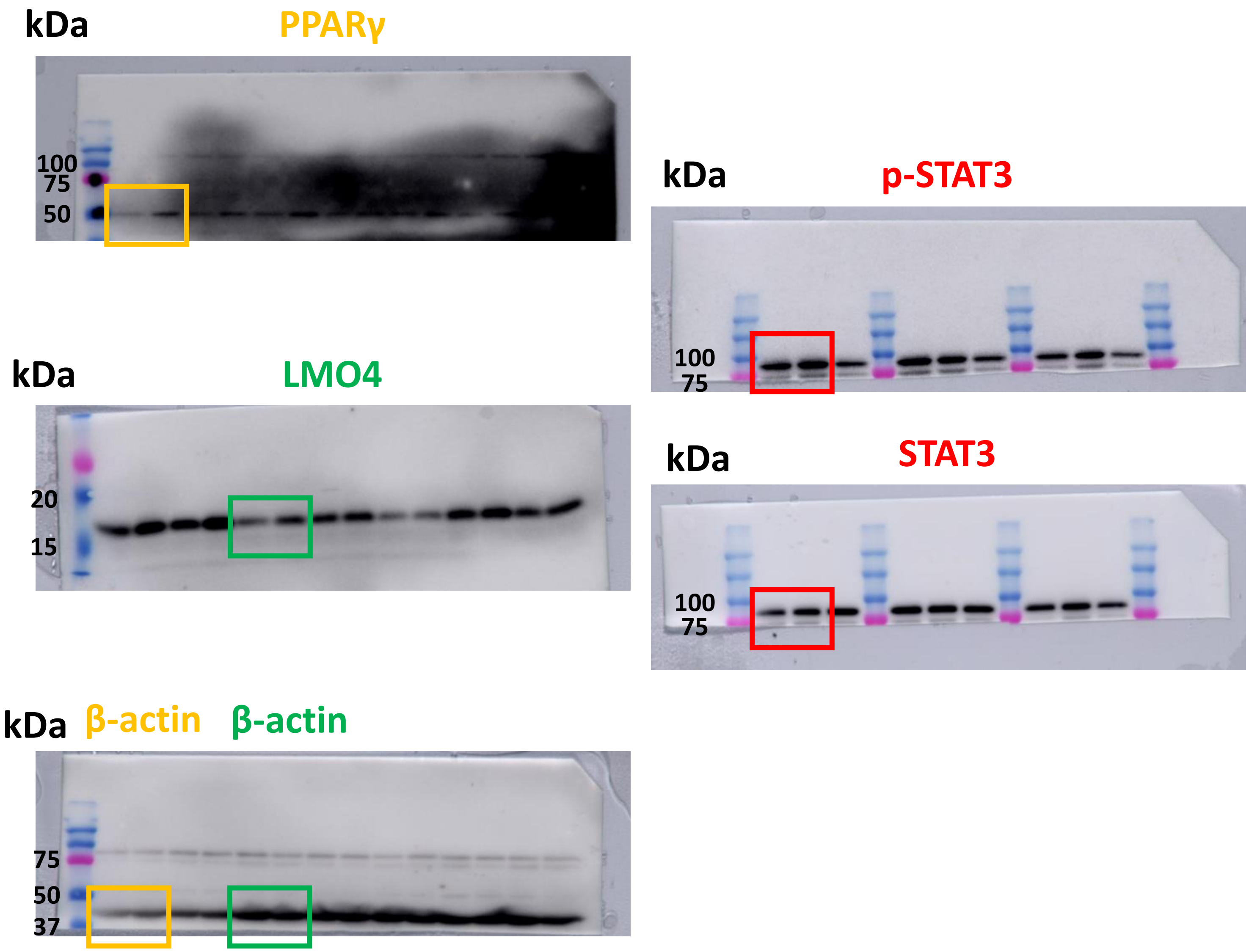

Supplementary Figure S7A. Unprocessed gels for Figure 4.

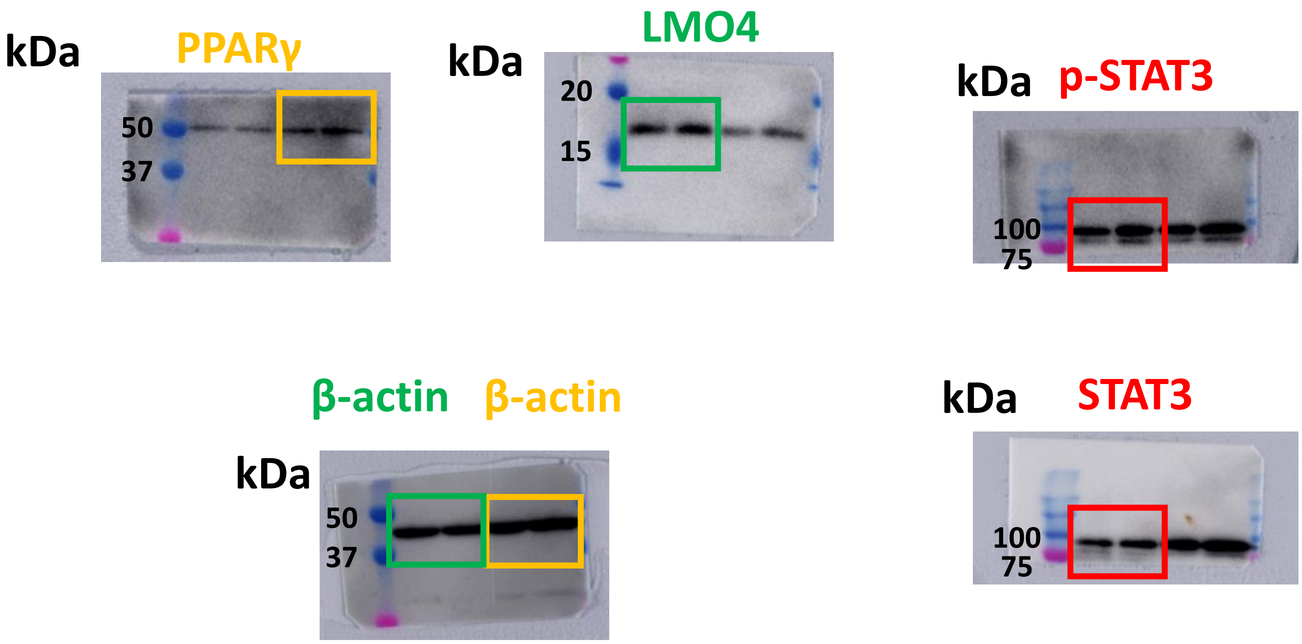

Supplementary Figure S7B. Unprocessed gels for Figure 6.

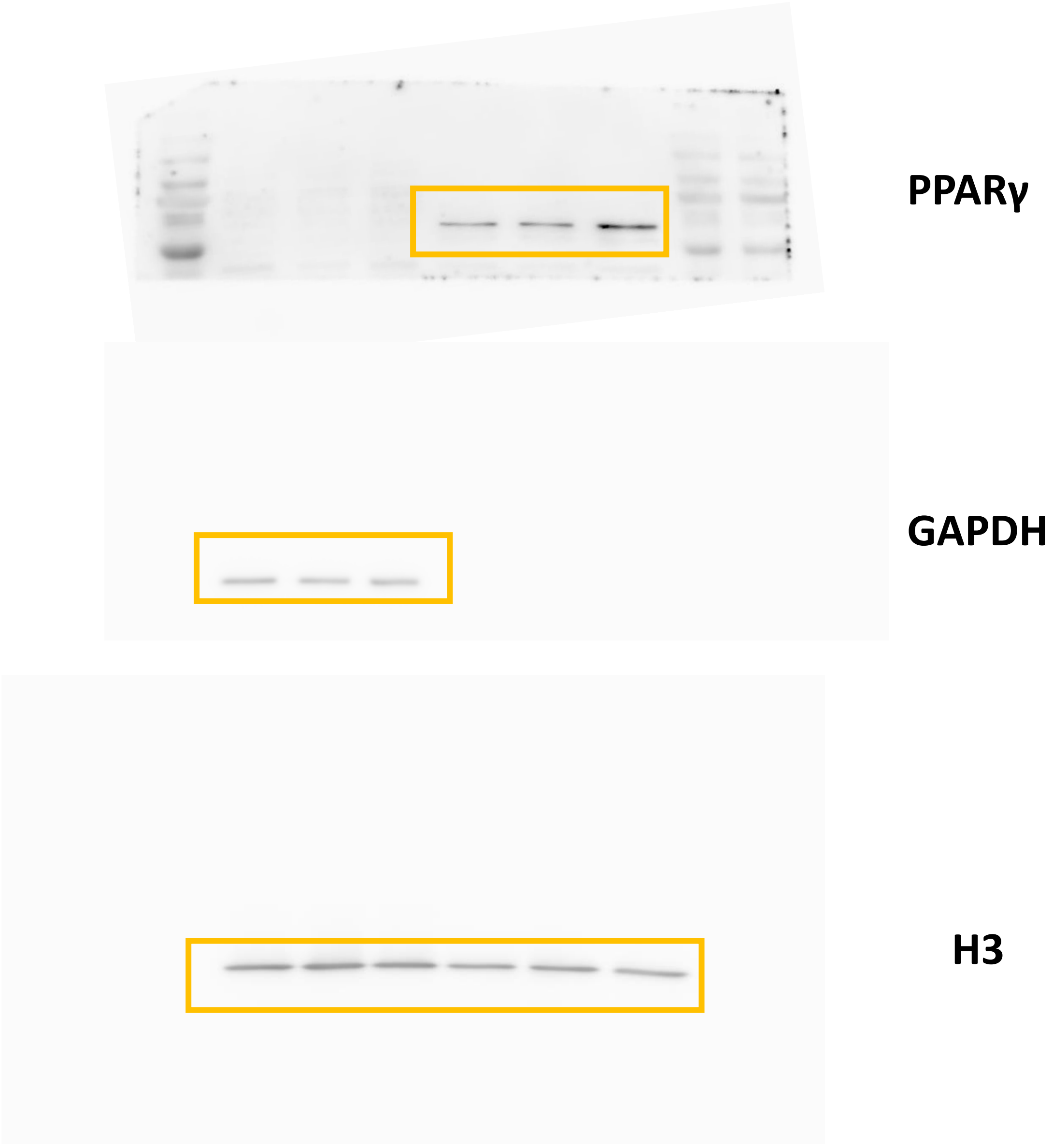

Supplementary Figure S7C. Unprocessed gels for Figure S3.

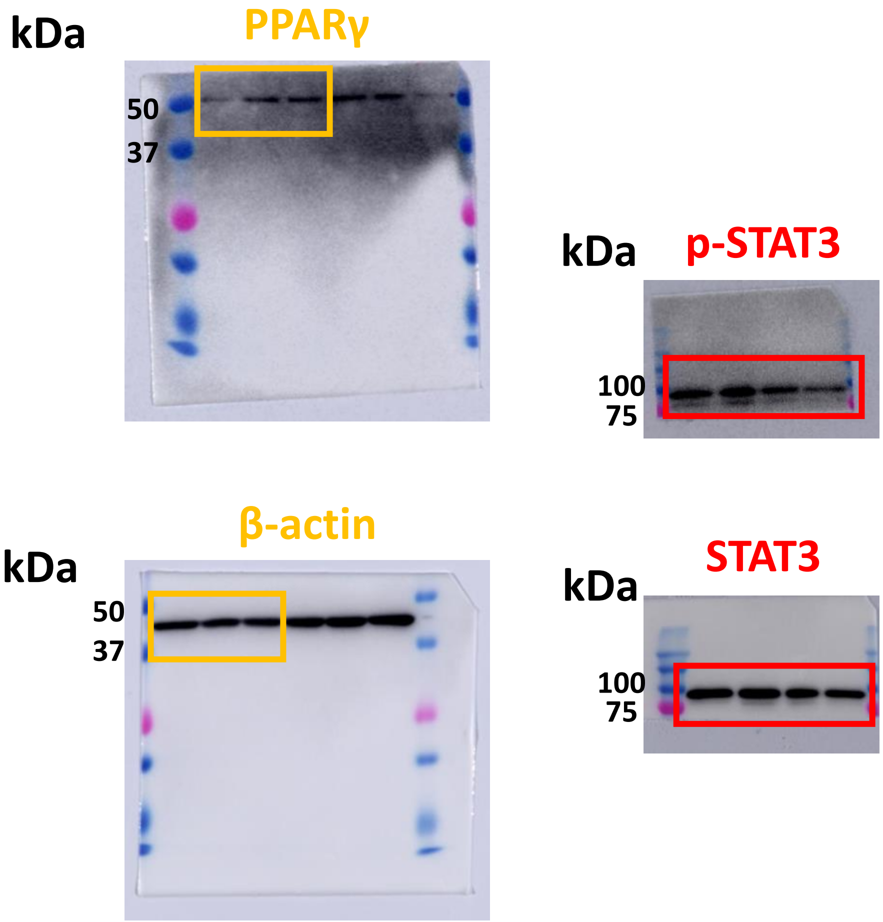

Supplementary Figure S7D. Unprocessed gels for Figure S4.
